# Supplementary material for: Somatic mutations in collagens are associated with a distinct tumor environment and overall survival in gastric cancer
Source: BMC Cancer. 2022 Feb 4;22:139. doi: 10.1186/s12885-021-09136-1 (PMC8815231; doi:10.1186/s12885-021-09136-1)
Supplement: Supplementary file 1 — Additional file 1 : Table S1. Collagen family for collagens of interest in stomach cancer. [file 12885_2021_9136_MOESM1_ESM.docx]

**Table S1. Collagen family for collagens of interest in stomach cancer**

| **Structural Family** | **Gene** | **Putative Role** | **Genetic Disease** | **References** |
| --- | --- | --- | --- | --- |
| **Fibril forming** | COL1A1 | Fiber collagen | Osteogenesis imperfecta; Ehlers-Danlos | (1) |
|  | COL1A2 | Fiber collagen | Osteogenesis imperfecta; Ehlers-Danlos | (1) |
|  | COL2A1 | Fiber collagen | spondyloepiphyseal dysplasia congenita | (2) |
|  | COL3A1 | Fiber collagen | Vascular Ehlers-Danlos | (3) |
|  | COL5A1, COL5A2 | Promotes Type I fibers | Classic Ehlers-Danlos | (4) |
|  | COL5A3 | Negative regulator of Type I fibers | N/A |  |
|  | COL11A1 | Promotes Type I fibers | Sickler Syndrome;  Marshall Syndrome; skeletal dysplasia | (5, 6) |
|  | COL11A2 | Promotes Type I fibers | Sickler Syndrome  Marshall Syndrome  Otospondylomegaepiphyseal dysplasia | (7) |
|  | COL14A1 | Fibril surface; Negative regulator of Type I fibers | Keratoderma | (8) |
|  | COL24A1 | Type I fibrillogenesis regulator |  |  |
|  | COL27A1 |  | Steel syndrome | (9) |
|  |  |  |  |  |
| **FACIT** | COL9A1 | Crosslinked to fibrils | Multiple epiphyseal dysplasia; osteoarthritis; Stickler syndrome | (7) |
|  | COL9A2 | Crosslinked to fibrils | multiple epiphyseal dysplasia | (7) |
|  | COL9A3 | Crosslinked to fibrils | Multiple epiphyseal dysplasia Autosomal recessive Stickler syndrome | (7) |
|  | COL12A1 | Basement membrane | myopathy | (10, 11) |
|  | COL15A1 | Banded Fibril linker | glaucoma | (12) |
|  | COL19A1 | Basement membrane zones |  |  |
|  | COL20A1 |  | N/A |  |
|  | COL21A1 |  | N/A |  |
|  | COL22A1 | Basement membrane zones | N/A |  |
| **Network** |  |  |  |  |
|  | COL4A1 | Basement | Familial porencephaly Hereditary angiopathy with nephropathy, aneurysms and muscle cramps syndrome | (13) |
|  | COL4A2 | Basement | Familial porencephaly Hereditary angiopathy with nephropathy, aneurysms and muscle cramps syndrome | (13) |
|  | COL4A3 | Basement | Alport Syndrome | (14) |
|  | COL4A4 | Basement | Alport Syndrome | (14) |
|  | COL4A5 | Basement | Alport Syndrome | (14) |
|  | COL4A6 | Basement | N/A |  |
|  | COL8A1 | Basement | N/A |  |
|  | COL10A1 | Chondrocyte matrix deposition | Schmid type metaphyseal chondrodysplasia; spondylometaphyseal dysplasia | (15) |
|  |  |  |  |  |
| **COL6** |  |  |  |  |
|  | COL6A1 | Basement membrane/interstitial matrix | Bethlem myopathy, Ullrich congenital muscular dystrophy | (16) |
|  | COL6A2, COL6A3 | Basement membrane/interstitial matrix | Ullrich congenital muscular dystrophy | (16) |
|  |  |  |  |  |
|  | COL7A1 | Dermoepidermal Anchoring fibril | Dystrophic epidermolysis bullosa (DEB) | (17) |
|  | COL26A1 |  | N/A |  |
|  | COL28A1 |  | N/A |  |
| **Membrane** | COL13A1 | Not known function | Congenital myasthenic | (18) |
|  | COL17A1 | Dermoepidermal anchoring complex | Junctional epidermolysis bullosa-other | (19) |
|  | COL23A1 | Not known function | N/A |  |
|  | COL25A1 | Linked with amyloid formation | congenital cranial dysinnervation disorder | (20) |
|  |  |  |  |  |
| **Multiplexins** | COL18A1 |  | Knobloch syndrome; glaucoma | (12) |

1. Marini JC, Forlino A, Bachinger HP, Bishop NJ, Byers PH, Paepe A, Fassier F, Fratzl-Zelman N, Kozloff KM, Krakow D, Montpetit K, Semler O. Osteogenesis imperfecta. Nat Rev Dis Primers. 2017;3:17052. Epub 2017/08/19. doi: 10.1038/nrdp.2017.52. PubMed PMID: 28820180.

2. Nenna R, Turchetti A, Mastrogiorgio G, Midulla F. COL2A1 Gene Mutations: Mechanisms of Spondyloepiphyseal Dysplasia Congenita. Appl Clin Genet. 2019;12:235-8. Epub 2019/12/12. doi: 10.2147/TACG.S197205. PubMed PMID: 31824186; PMCID: PMC6900288.

3. Kuivaniemi H, Tromp G. Type III collagen (COL3A1): Gene and protein structure, tissue distribution, and associated diseases. Gene. 2019;707:151-71. Epub 2019/05/11. doi: 10.1016/j.gene.2019.05.003. PubMed PMID: 31075413; PMCID: PMC6579750.

4. Malfait F, De Paepe A. Molecular genetics in classic Ehlers-Danlos syndrome. Am J Med Genet C Semin Med Genet. 2005;139C(1):17-23. Epub 2005/11/10. doi: 10.1002/ajmg.c.30070. PubMed PMID: 16278879.

5. Martin S, Richards AJ, Yates JR, Scott JD, Pope M, Snead MP. Stickler syndrome: further mutations in COL11A1 and evidence for additional locus heterogeneity. Eur J Hum Genet. 1999;7(7):807-14. Epub 1999/11/26. doi: 10.1038/sj.ejhg.5200377. PubMed PMID: 10573014.

6. Annunen S, Korkko J, Czarny M, Warman ML, Brunner HG, Kaariainen H, Mulliken JB, Tranebjaerg L, Brooks DG, Cox GF, Cruysberg JR, Curtis MA, Davenport SL, Friedrich CA, Kaitila I, Krawczynski MR, Latos-Bielenska A, Mukai S, Olsen BR, Shinno N, Somer M, Vikkula M, Zlotogora J, Prockop DJ, Ala-Kokko L. Splicing mutations of 54-bp exons in the COL11A1 gene cause Marshall syndrome, but other mutations cause overlapping Marshall/Stickler phenotypes. Am J Hum Genet. 1999;65(4):974-83. Epub 1999/09/16. doi: 10.1086/302585. PubMed PMID: 10486316; PMCID: PMC1288268.

7. Carter EM, Raggio CL. Genetic and orthopedic aspects of collagen disorders. Curr Opin Pediatr. 2009;21(1):46-54. Epub 2009/03/04. doi: 10.1097/mop.0b013e32832185c5. PubMed PMID: 19253462.

8. Guo BR, Zhang X, Chen G, Zhang JG, Sun LD, Du WD, Zhang Q, Cui Y, Zhu J, Tang XF, Xiao R, Liu Y, Li M, Tang HY, Yang X, Cheng H, Li M, Gao M, Li P, Wang JB, Xu FP, Zuo XB, Zheng XD, Zhang XG, Yang L, Liu JJ, Wang J, Yang S, Zhang XJ. Exome sequencing identifies a COL14A1 mutation in a large Chinese pedigree with punctate palmoplantar keratoderma. J Med Genet. 2012;49(9):563-8. Epub 2012/09/14. doi: 10.1136/jmedgenet-2012-100868. PubMed PMID: 22972947.

9. Evie K, Athina T, Nayia N, Angelos A, Ioannis P, Elisavet E, Violetta C-A, Carolina S, Tanteles GA. First reported case of Steel syndrome in the European population: A novel homozygous mutation in COL27A1 and review of the literature. Eur J Med Genet. 2020;63(7):103939. Epub 2020/05/04. doi: 10.1016/j.ejmg.2020.103939. PubMed PMID: 32360765.

10. Hicks D, Farsani GT, Laval S, Collins J, Sarkozy A, Martoni E, Shah A, Zou Y, Koch M, Bonnemann CG, Roberts M, Lochmuller H, Bushby K, Straub V. Mutations in the collagen XII gene define a new form of extracellular matrix-related myopathy. Hum Mol Genet. 2014;23(9):2353-63. Epub 2013/12/18. doi: 10.1093/hmg/ddt637. PubMed PMID: 24334769.

11. Zou Y, Zwolanek D, Izu Y, Gandhy S, Schreiber G, Brockmann K, Devoto M, Tian Z, Hu Y, Veit G, Meier M, Stetefeld J, Hicks D, Straub V, Voermans NC, Birk DE, Barton ER, Koch M, Bonnemann CG. Recessive and dominant mutations in COL12A1 cause a novel EDS/myopathy overlap syndrome in humans and mice. Hum Mol Genet. 2014;23(9):2339-52. Epub 2013/12/18. doi: 10.1093/hmg/ddt627. PubMed PMID: 24334604; PMCID: PMC3976332.

12. Wiggs JL, Howell GR, Linkroum K, Abdrabou W, Hodges E, Braine CE, Pasquale LR, Hannon GJ, Haines JL, John SW. Variations in COL15A1 and COL18A1 influence age of onset of primary open angle glaucoma. Clin Genet. 2013;84(2):167-74. Epub 2013/04/30. doi: 10.1111/cge.12176. PubMed PMID: 23621901; PMCID: PMC3771394.

13. Kuo DS, Labelle-Dumais C, Gould DB. COL4A1 and COL4A2 mutations and disease: insights into pathogenic mechanisms and potential therapeutic targets. Hum Mol Genet. 2012;21(R1):R97-110. doi: 10.1093/hmg/dds346. PubMed PMID: 22914737; PMCID: PMC3459649.

14. Savige J, Harraka P. Pathogenic Variants in the Genes Affected in Alport Syndrome (COL4A3-COL4A5) and Their Association With Other Kidney Conditions: A Review. Am J Kidney Dis. 2021. Epub 2021/07/11. doi: 10.1053/j.ajkd.2021.04.017. PubMed PMID: 34245817.

15. Bateman JF, Freddi S, McNeil R, Thompson E, Hermanns P, Savarirayan R, Lamande SR. Identification of four novel COL10A1 missense mutations in schmid metaphyseal chondrodysplasia: further evidence that collagen X NC1 mutations impair trimer assembly. Hum Mutat. 2004;23(4):396. Epub 2004/03/17. doi: 10.1002/humu.9222. PubMed PMID: 15024737.

16. Lamande SR, Bateman JF. Collagen VI disorders: Insights on form and function in the extracellular matrix and beyond. Matrix Biol. 2018;71-72:348-67. Epub 2017/12/27. doi: 10.1016/j.matbio.2017.12.008. PubMed PMID: 29277723.

17. Wertheim-Tysarowska K, Sobczynska-Tomaszewska A, Kowalewski C, Skronski M, Swieckowski G, Kutkowska-Kazmierczak A, Wozniak K, Bal J. The COL7A1 mutation database. Hum Mutat. 2012;33(2):327-31. Epub 2011/11/08. doi: 10.1002/humu.21651. PubMed PMID: 22058051.

18. Rodriguez Cruz PM, Cossins J, Estephan EP, Munell F, Selby K, Hirano M, Maroofin R, Mehrjardi MYV, Chow G, Carr A, Manzur A, Robb S, Munot P, Wei Liu W, Banka S, Fraser H, De Goede C, Zanoteli E, Conti Reed U, Sage A, Gratacos M, Macaya A, Dusl M, Senderek J, Topf A, Hofer M, Knight R, Ramdas S, Jayawant S, Lochmuller H, Palace J, Beeson D. The clinical spectrum of the congenital myasthenic syndrome resulting from COL13A1 mutations. Brain. 2019;142(6):1547-60. Epub 2019/05/14. doi: 10.1093/brain/awz107. PubMed PMID: 31081514; PMCID: PMC6752227.

19. Has C, Kern JS. Collagen XVII. Dermatol Clin. 2010;28(1):61-6. Epub 2009/12/01. doi: 10.1016/j.det.2009.10.007. PubMed PMID: 19945617.

20. Shinwari JM, Khan A, Awad S, Shinwari Z, Alaiya A, Alanazi M, Tahir A, Poizat C, Al Tassan N. Recessive mutations in COL25A1 are a cause of congenital cranial dysinnervation disorder. Am J Hum Genet. 2015;96(1):147-52. Epub 2014/12/17. doi: 10.1016/j.ajhg.2014.11.006. PubMed PMID: 25500261; PMCID: PMC4289688.
