# Supplementary material for: Somatic mutations in collagens are associated with a distinct tumor environment and overall survival in gastric cancer
Source: BMC Cancer. 2022 Feb 4;22:139. doi: 10.1186/s12885-021-09136-1 (PMC8815231; doi:10.1186/s12885-021-09136-1)
Supplement: Supplementary file 2 — Additional file 2 : Table S2. MutSig 2CV v3.1 analysis of significantly mutated collagen genes in STAD TCGA cohort. Data downloaded from Firebrowse. [file 12885_2021_9136_MOESM2_ESM.docx]

**Supplemental Table S2. Collagen genes significantly mutated in the TCGA STAD cohort as assessed by MutSigCV2.**

| **Gene** | **p-value** | **q-value** |
| --- | --- | --- |
| **COL12A1** | **1.1e-4** | **0.006** |
| **COL11A1** | **0.001** | **0.04** |
| **COL20A1** | **0.004** | **0.09** |
| **COL8A1** | **0.014** | **0.2** |
